# Potential of lactoferrin to prevent antibiotic-induced *Clostridium difficile* infection

**DOI:** 10.1093/jac/dkv452

**Published:** 2016-01-11

**Authors:** C. H. Chilton, G. S. Crowther, K. Śpiewak, M. Brindell, G. Singh, M. H. Wilcox, T. M. Monaghan

**Affiliations:** 1 Leeds Institute for Molecular Medicine, University of Leeds, Leeds, UK; 2 Department of Inorganic Chemistry, Jagiellonian University, Krakow, Poland; 3 NIHR Biomedical Research Unit in Gastrointestinal and Liver Diseases at Nottingham University Hospitals NHS Trust and The University of Nottingham, Nottingham, UK

## Abstract

**Objectives:**

*Clostridium difficile* infection (CDI) is a global healthcare problem. Recent evidence suggests that the availability of iron may be important for *C. difficile* growth. This study evaluated the comparative effects of iron-depleted (1% Fe^3+^ saturated) bovine apo-lactoferrin (apo-bLf) and iron-saturated (85% Fe^3+^ saturated) bovine holo-lactoferrin (holo-bLf) in a human *in vitro* gut model that simulates CDI.

**Methods:**

Two parallel triple-stage chemostat gut models were inoculated with pooled human faeces and spiked with *C. difficile* spores (strain 027 210, PCR ribotype 027). Holo- or apo-bLf was instilled (5 mg/mL, once daily) for 35 days. After 7 days, clindamycin was instilled (33.9 mg/L, four times daily) to induce simulated CDI. Indigenous microflora populations, *C. difficile* total counts and spores, cytotoxin titres, short chain fatty acid concentrations, biometal concentrations, lactoferrin concentration and iron content of lactoferrin were monitored daily.

**Results:**

In the apo-bLf model, germination of *C. difficile* spores occurred 6 days post instillation of clindamycin, followed by rapid vegetative cell proliferation and detectable toxin production. By contrast, in the holo-bLf model, only a modest vegetative cell population was observed until 16 days post antibiotic administration. Notably, no toxin was detected in this model. In separate batch culture experiments, holo-bLf prevented *C. difficile* vegetative cell growth and toxin production, whereas apo-bLf and iron alone did not.

**Conclusions:**

Holo-bLf, but not apo-bLf, delayed *C. difficile* growth and prevented toxin production in a human gut model of CDI. This inhibitory effect may be iron independent. These observations suggest that bLf in its iron-saturated state could be used as a novel preventative or treatment strategy for CDI.

## Introduction

*Clostridium difficile* infection (CDI) is a global healthcare problem. It is the leading cause of hospital-acquired infectious diarrhoea, causing significant morbidity and societal financial burden.^1,2^ Recurrent infections and increasing antibiotic resistance have complicated the treatment of CDI.^3–5^ There is now, therefore, an urgent need for the development of novel, non-antibiotic-based therapeutic and preventative strategies. To successfully sustain an infection, nearly all bacteria, fungi and protozoa require a continuous supply of host iron, which participates in many essential metabolic processes.^6^ Recent *in vitro* evidence suggests that the availability and source of iron may be important for *C. difficile* growth.^7^ The development of pharmaceutical agents that can manipulate microbial access to iron may help prevent and possibly treat CDI.

Lactoferrin, a multifunctional ferric (Fe^3+^) iron-binding glycoprotein found in milk, in several mucosal secretions and in secondary granules of neutrophils, exhibits bacteriostatic activity due to its ability to sequester iron, thereby depriving potential pathogens of this essential nutrient.^8^ Multiple *in vitro* and animal studies show that both human and bovine iron-depleted forms of lactoferrin, which share similar sequence homology, similar three-dimensional structures and comparable bioactivity, inhibit growth of enteric bacteria.^9^ Faecal lactoferrin is a biomarker for intestinal inflammation.^10^ Quantitative faecal lactoferrin has been shown to correlate with the degree of colonic inflammation and disease severity in inflammatory bowel disease.^10–12^ Similarly, faecal lactoferrin concentration has been shown to be elevated in CDI patients,^13–15^ with more recent reports suggesting a positive correlation with disease severity.^13–16^ However, it is acknowledged that there is wide variability among patients and that larger cohort studies are needed in order to evaluate the potential of faecal lactoferrin concentration as an objective predictor of poor outcome in hospitalized patients.^15^

The aim of this research was to evaluate for the first time the comparative effects of iron-depleted (1% Fe^3+^ saturated) bovine apo-lactoferrin (apo-bLf) and iron-saturated (85% Fe^3+^ saturated) bovine holo-lactoferrin (holo-bLf) in a human *in vitro* gut model predictive of CDI. We hypothesized that apo-bLf (unlike holo-bLf) would compete with *C. difficile* for free iron, thus inhibiting *C. difficile* growth, sporulation and toxin production and so preventing CDI while having little effect on the indigenous gut microbiota.

## Materials and methods

### Triple-stage *in vitro* human gut model

A validated and clinically reflective triple-stage chemostat model was used to simulate CDI. The model consists of three water-jacketed (37°C) glass vessels arranged in a weir cascade formation and top fed with a complex growth media (dilution rate, ∼13.2 mL/h). The model is primed with a pooled human faecal emulsion, and each vessel is sparged with nitrogen to ensure anaerobic conditions and maintained at a gut-reflective pH (vessel 1 = 5.5, vessel 2 = 6.2, vessel 3 = 6.8). The system has been validated against the intestinal contents of sudden-death victims, and it provides a close simulation of bacterial activities and composition in different areas of the colon.

### *C. difficile* strain and spore preparation

*C. difficile* strain 027 210 (BI/NAP1/PCR ribotype 027/ toxinotype III) was used. This strain was isolated during a clinical outbreak of CDI at Maine Medical Center (Portland, ME, USA) and was kindly supplied by Dr Robert Owens. Spores were initially grown on cefoxitin/cycloserine/egg yolk agar plus lysozyme (CCEY/L) for 48 h, before being subbed onto Columbia Blood Agar (40 plates). Following checks for purity, all growth was harvested into sterile saline (2 mL). An equal volume of ethanol was added to the spore suspension to kill any vegetative cells. The spore suspension was subject to trypsin digestion and washing, to remove debris, and enumerated on CCEYL. The spore preparation was diluted in 50% ethanol to achieve ∼10^7^ cfu/mL.

### Preparation of bovine apo- and holo-lactoferrins

Bovine lactoferrin (bLf) was purchased from Morinaga Milk Industry (80% purity and 10% Fe^3+^ saturation; Zama, Japan) and stored at 4°C. Apo-lactoferrin (apo-bLf) was prepared by dissolution of lactoferrin powder in Milli-Q water to obtain 50 mg/mL solution and dialysed extensively against 100 mM citrate buffer (POCH S.A.; Gliwice, Poland), pH 3.5 with 200-fold volume excess of the buffer for 24 h (with three buffer changes), followed by dialysis against Milli-Q water for 24 h.^17^ Holo-lactoferrin (holo-bLf) was prepared by the reaction of 50 mg/mL lactoferrin solution in 50 mM Tris-(hydroxymethyl) aminomethane (VWR BDH Prolabo; Dublin, Ireland), 150 mM sodium chloride (POCH S.A., Gliwice, Poland), 25 mM sodium bicarbonate (pH 7.4; Sigma-Aldrich) with 4-fold molar excess of ferric nitrate salt over lactoferrin. Buffers and reagents in all experiments were prepared using Milli-Q water (18 MΩ). After 1 h of incubation at room temperature, unbound iron was removed by dialysis against Milli-Q water for 24 h (with three water changes).^17^ Both apo- and holo-bLf were freeze dried and stored at 4°C. Iron saturation of apo- and holo-bLf preparations was calculated based on the A280/A466 ratio according to the calibration curve presented by Majka *et al.*^17^ Prepared apo-bLf was 1% Fe^3+^ saturated, while holo-bLf Fe^3+^ saturation was 85%.

### Experimental design

**Figure 1.**
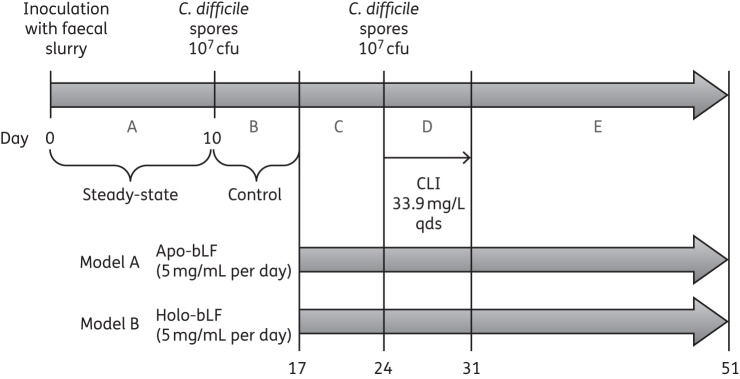
Experimental design. A = steady-state, B = *C. difficile* control period, C = lactoferrin control period, D = *C. difficile* infection and E = rest stage. CLI, clindamycin; qds, four times daily.

Two models were run in parallel (Figure 1). Each vessel was primed with ∼150 mL of faecal emulsion (10% w/v in pre-reduced PBS) prepared from *C. difficile*-negative faecal samples from five healthy volunteers (age >60 years) with no history of antibiotic therapy for >3 months. The volunteers gave appropriate consent for their samples to be used in this study. The models were left for 10 days to equilibrate to allow bacterial populations to reach steady-state (period A). A single aliquot of *C. difficile* PCR ribotype 027 spores (∼10^7^ cfu) was added into vessel 1 of each model and left for a 7 day *C. difficile* control period (period B). Lactoferrin instillation commenced on day 17 and continued for the duration of the experiment. 5 mg/mL apo-bLf was added once daily to model A and 5 mg/mL holo-bLf was added once daily to model B. After a 7 day lactoferrin control period (period C), a second aliquot of *C. difficile* spores was added and clindamycin instillation commenced (33.9 mg/L, four times daily for 7 days, period D). Following clindamycin instillation, the models were monitored for a further 3 weeks (period E). Gut microflora populations in vessels 2 and 3 were monitored on selective and non-selective agars on the basis of appearance, colony morphology, microscopy and MALDI-TOF, as previously described,^18^ every other day during period A and daily thereafter. *C. difficile* total viable counts (vegetative cells and spores), spore counts and toxin titres in all vessels were enumerated daily from period B onwards, as previously described.^18^ Briefly, toxin levels were measured by cell cytotoxicity assay daily following inoculation of *C. difficile* into the gut model. Model fluid was centrifuged, and the supernatant was subjected to 10-fold dilution. Diluted supernatant was inoculated onto confluent Vero cells and incubated, and cells were examined after 24 and 48 h. Samples were considered positive for *C. difficile* toxin if cell rounding was observed in 80% of wells and effects were neutralized by the presence of sordelli antitoxin. If rounding was observed in the neat sample only, a titre of 1 relative unit (RU) was assigned; a titre of 2 RU was assigned if cell rounding was observed in neat and 1:10 dilutions and a titre of 4 RU was assigned if cell rounding was observed in neat, 1:10 and 1:100 dilutions.

### Ethics statement

Ethics approval for the collection and use of faecal donations from healthy adult volunteers was provided by the Leeds Institute of Health Sciences and Leeds Institute of Genetics, Health and Therapeutics and Leeds Institute of Molecular Medicine, University of Leeds joint ethics committee (reference HSLTLM/12/061).

### Determination of lactoferrin concentrations

The concentration of lactoferrin in the gut-model fluid was measured using a bLf ELISA kit (Alpha Diagnostic International, San Antonio, TX, USA). Representative samples were drawn at 19 pre-selected timepoints across the different experimental time periods following addition of lactoferrin. A standard curve was prepared with the supplied bLf standards with concentrations ranging from 4 to 80 ng/mL. The analysed gut-model fluid samples were diluted 1:100 000 in sample diluent buffer and the test was performed according to the manufacturer's protocol. ELISA plates were read using an Infinite 200 Reader (Tecan) plate at 450 nm to determine lactoferrin concentration.

### Estimation of iron saturation in lactoferrin preparations

Lactoferrin saturation was evaluated in the high molecular fraction of gut-model fluid samples at 12 separate timepoints. These points again were chosen as representative timepoints following addition of lactoferrin. Protein content in test samples before and after lactoferrin application was analysed by SDS-PAGE. Prior to lactoferrin application, no proteins were detected in the gut-model fraction; after lactoferrin addition, the major fraction was lactoferrin. Iron concentration in fractions higher than 3 kDa was attributed to lactoferrin. To separate lactoferrin bound iron and free iron, 500 μL test samples were ultrafiltered on Amicon Ultra-4 Centrifugal Filter Units (Merck Millipore) with molecular weight cut-off 3 kDa (7500 g, 30 min, 4°C). Lactoferrin concentration was analysed using ELISA in the high molecular fraction, following a 1 000 000× dilution of the tested samples with the diluent buffer. Iron content was analysed after mineralization of 40 μL of test sample with 500 μL of concentrated nitric acid (65%, Suprapur) overnight. Iron concentration was determined in these mineralized samples following dilution with Milli-Q water to obtain a 5 mL solution using the inductively coupled plasma optical emission spectroscopy (ICP-OES) technique. The latter was performed with a Plasma 40 PerkinElmer spectrometer. The ^57^Fe isotope was used for the quantification of iron content in samples.

### Determination of antimicrobial concentrations

Gut-model fluid was sampled daily from each vessel from the start of period D onwards, and clindamycin concentrations were determined by large-plate bioassay. Samples were frozen at −20°C and assayed retrospectively. Defrosted samples were centrifuged, and the supernatants were filtered through 0.22 μm syringe filters. Wilkins–Chalgren agar (100 mL) was sterilized by autoclaving, cooled to 50°C, inoculated with 1 mL *Kocuria rhizophila* (ATCC 9341) indicator organism suspension and transferred aseptically into 245 × 245 mm agar plates. Plates were dried (37°C, 10 min) and 25 wells dug with a 9 mm diameter cork borer. Twenty microlitres of antibiotic calibrator or gut-model sample was inoculated into bioassay wells. Bioassay plates were incubated at 37°C overnight. Diameters of each zone of inhibition were measured using callipers accurate to 0.1 mm, and antimicrobial concentrations were determined by comparison with known antimicrobial standard concentrations.

### Determination of biometal concentrations

Total iron, zinc, copper and manganese biometal content in vessel 3 gut-model samples was measured by the application of the ICP-OES technique using a Plasma 40 PerkinElmer spectrometer. Representative samples were drawn at 19 timepoints, representing all experimental time periods. Prior to the determination of biometal content, 100 μL of the tested samples was incubated overnight with 500 μL of concentrated nitric acid and subsequently diluted with Milli-Q water to obtain a 5 mL solution. Metal content in the samples was measured with two repetitions.

### Determination of short chain fatty acid (SCFA) concentrations

SCFA concentrations (μM) of the gut-model fluid samples in vessel 3 were quantified at 20 representative timepoints spanning the entire experimental period by GC-MS. Standard solutions of acetic acid, propanoic acid, butyric acid, isobutyric acid, valeric acid and isovaleric acid were prepared at different concentrations (100, 200, 400 and 800 μM). Injection was made in splitless mode with an injection volume of 1 μL. The volatiles were transferred onto a new Zebron ZB-FFAP column (30 m × 0.25 mm × 0.25 μm film thickness; Phenomenex, UK) and chromatogram with helium as the carrier gas at 18 psi. The GC starting temperature was 60°C, held for 1 min and ramped to 180°C at 8°C/min. All the compounds were detected with a DSQ mass spectrometer (Thermo Scientific) in scan mode, 20–150 mass:charge ratio). Identification of SCFAs was based on the retention time of standard compounds and with the assistance of the data library of the National Institute of Standards and Technology.

### Lactoferrin batch culture

Aliquots of brain heart infusion (BHI) broth (10 mL) in glass vials were supplemented with either holo-bLf (50 mg), apo-bLf (50 mg) or iron (50 μL of stock solution containing 496 mg of FeO_4_S·7H_2_O in 10 mL of H_2_O) alongside non-supplemented controls. These broths were inoculated with *C. difficile* (strain 027 210). One set of broths was inoculated with 100 μL of *C. difficile* spores (∼10^8^ cfu/mL) and the other set of broths was inoculated with 100 μL of an overnight vegetative Schaedler broth culture. Samples were taken at 24 and 48 h, and total viable counts, spore counts and toxin titres were enumerated in triplicate (as previously described).

## Results

### *C. difficile* total viable counts, spore counts and cytotoxin

**Figure 2.**
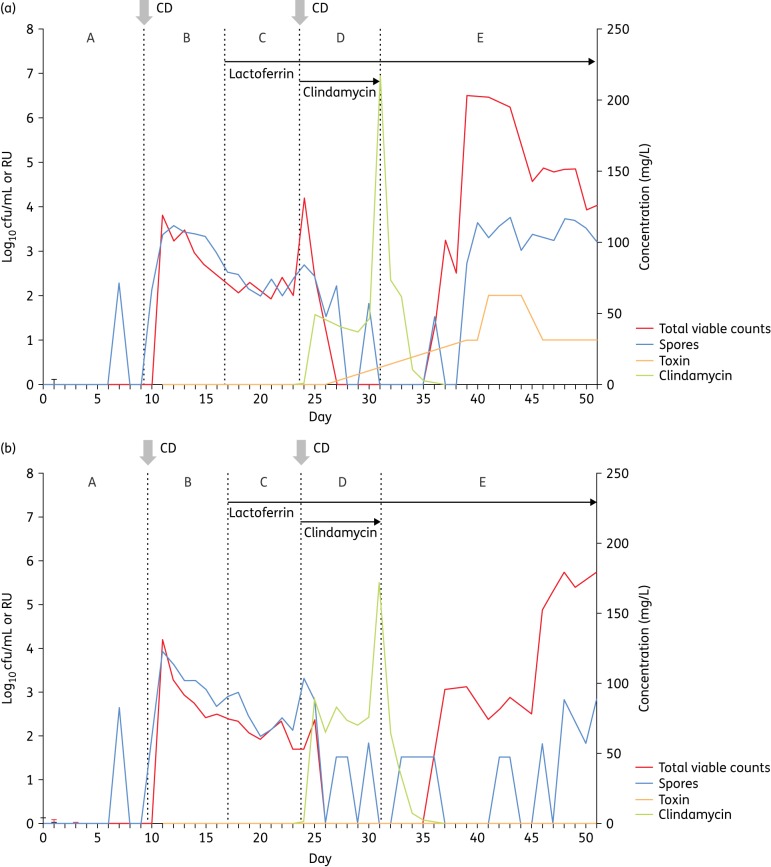
Planktonic *C. difficile* populations in vessel 3 of the (a) apo-lactoferrin and (b) holo-lactoferrin models. CD indicates insertion of *C. difficile* spores. A–E indicate experimental stages (see the Materials and methods section). This figure appears in colour in the online version of *JAC* and in black and white in the print version of *JAC*.

As seen in previous gut-model experiments, *C. difficile* remained as spores before (period B) and during (period C) clindamycin instillation in both models (Figure 2). Following the wash-out of detectable clindamycin activity (∼4 days into period E), an increase in total viable counts over spore counts was observed, indicating a vegetative cell population. In the apo-bLf model, this germination was followed by rapid vegetative cell proliferation and toxin production (Figure 2a). However, in the holo-bLf model, there was only a modest vegetative cell population (total counts ∼1 log_10_ cfu/mL greater than spore counts), with prolific vegetative growth not occurring until 16 days post clindamycin instillation. Notably, no toxin was detected in the holo-bLf model at any timepoint (Figure 2b).

### Gut microflora counts

The effect of clindamycin instillation (period C) on gut microflora populations was similar in both models and similar to that in previously described gut-model work (data not shown).^18–21^ *Bifidobacteria* populations decreased ∼7 log_10_ cfu/mL, to below the level of detection, but recovered to steady-state levels by the end of the experiment. Enterococci populations increased ∼4 log_10_ cfu/mL. Minor fluctuations were observed in other populations, but population stability returned by the end of the experiment.

### Lactoferrin and iron-saturation levels

**Figure 3.**
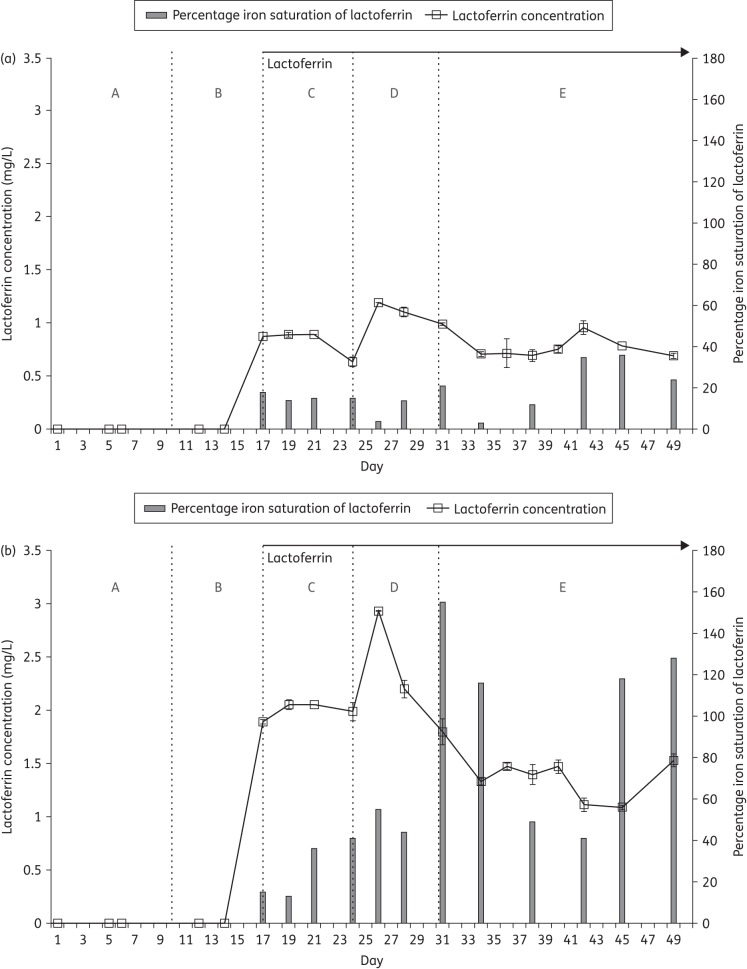
Lactoferrin concentration and iron saturation in vessel 3 of the (a) apo-lactoferrin and (b) holo-lactoferrin models. A–E indicate experimental stages (see the Materials and methods section).

Lower mean concentrations of lactoferrin (∼1 mg/mL) were observed in vessel 3 of the apo-bLf model from period C onwards compared with concentrations in the holo-bLf parallel model (2 mg/mL rising to a peak of just under 3 mg/mL (Figure 3). These findings suggest that both forms of lactoferrin may be subject to proteolytic degradation, with apo-bLf showing greater susceptibility than holo-bLf. Alternatively, lactoferrin may be being absorbed on the bacterial cell walls or protein precipitation may account for the lower levels of bLf in both models. The iron content of lactoferrin was higher in the iron-laden holo-bLf model rising to a peak of just under 160% following clindamycin instillation and CDI induction (Figure 3b). Iron-saturation values of >100% indicate that there is more iron relative to accessible iron-binding sites in lactoferrin. These iron ions can be bound in a non-specific way as trivalent cations.

### Total metal concentrations

Total iron concentration was higher in the iron-laden holo-bLf model, rising to a peak of just under 10 mg/L compared with that in the parallel apo-bLf model, in which levels peaked at just over 1 mg/L (data not shown). No marked differences in the concentrations of the other total metals were observed between the models, in which manganese and zinc concentrations were just above the limits of detection, unlike that of copper, which fell below the limit of detection (data not shown).

### SCFA concentrations

**Figure 4.**
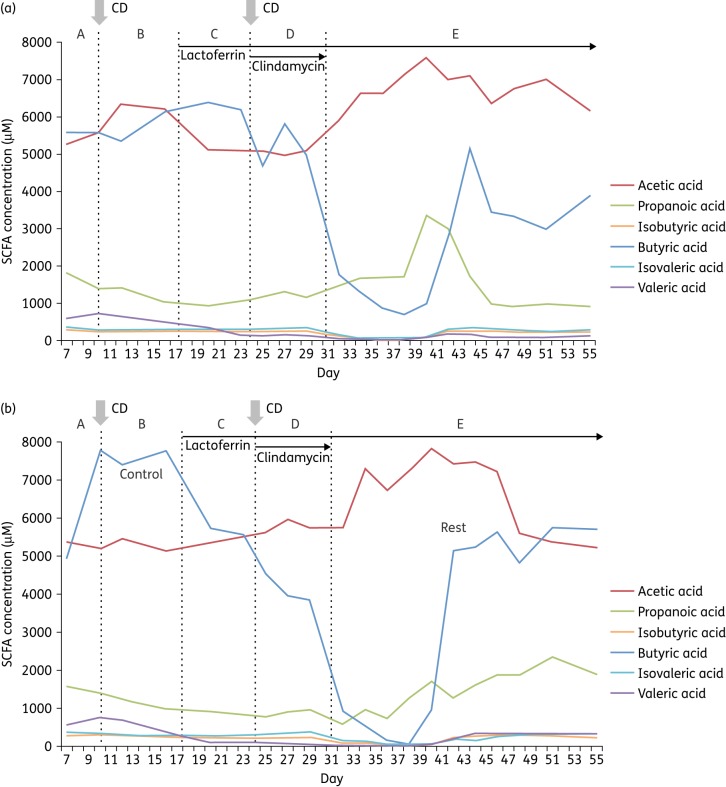
SCFA concentration in vessel 3 of the (a) apo-lactoferrin and (b) holo-lactoferrin models by GC-MS. CD indicates insertion of *C. difficile* spores. A–E indicate experimental stages (see the Materials and methods section). This figure appears in colour in the online version of *JAC* and in black and white in the print version of *JAC*.

Acetic acid, propanoic acid, isobutyric acid, butyric acid, isovaleric acid and valeric acid were present in varying concentrations in all samples tested from vessel 3 of each gut model. Both acetic and butyric acids were the most quantitatively significant SCFAs produced (Figure 4), followed by propanoic, isovaleric, valeric and isobutyric acids. Throughout, concentrations of valeric, isovaleric and isobutyric acids were very low. Clindamycin-induced disruption of gut-model-associated microbial communities was accompanied by a sharp decline in butyric acid production, which recovered in the rest phase, although recovery in the holo-bLf model was 3 days later and only partial (Figure 4b). In both models, acetic acid concentration increased following antibiotic administration.

### Investigation of the effects of holo-bLf, apo-bLf and additional iron (iron sulphate) on *C. difficile* growth, sporulation and toxin production when cultured using *in vitro* batch culture

Iron concentrations in all samples did not vary greatly between 24 and 48 h of incubation. Control and apo-bLf-supplemented broths contained ∼1–4 mg/L iron. Holo-bLf-supplemented broths contained ∼13–19 mg/L iron (similar to the ∼10 mg/L observed in the holo-bLf gut model) and the iron-supplemented broths contained ∼35–38 mg/L iron, exceeding those concentrations observed in the holo-bLf gut model.

**Figure 5.**
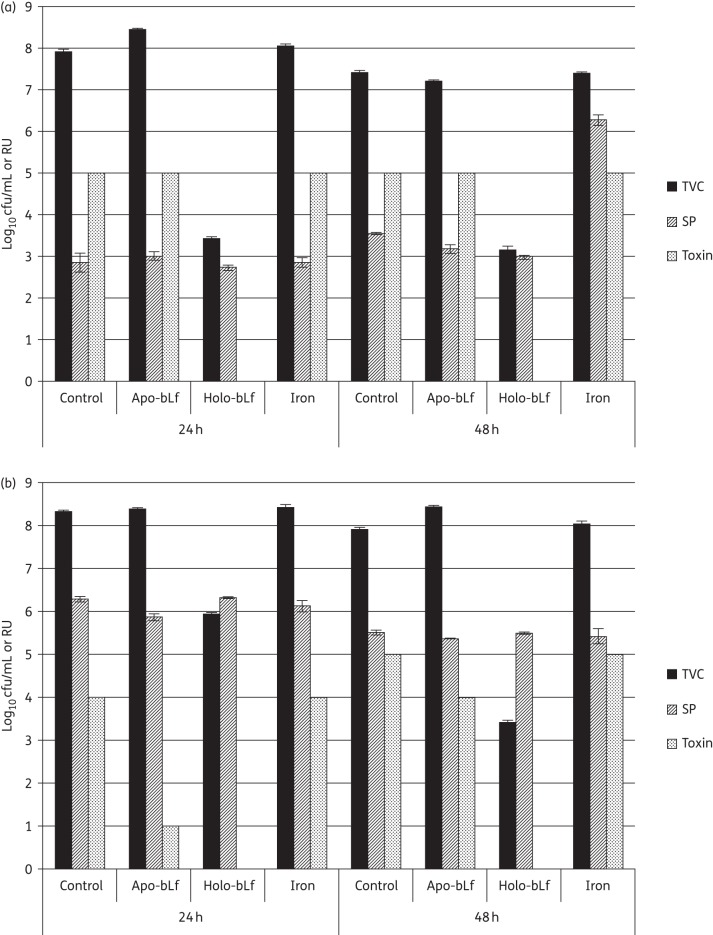
*C. difficile* total viable counts (TVC; in log_10_ cfu/mL), spore counts (SP; in log_10_ cfu/mL) and toxin titre (in RU) at 24 and 48 h following inoculation with (a) vegetative *C. difficile* and (b) *C. difficile* spores into non-supplemented BHI (control) or BHI supplemented with apo-lactoferrin (apo-bLf), holo-lactoferrin (holo-bLf) or iron. Bars represent ±SEM.

Following inoculation with *C. difficile* overnight culture, high levels of vegetative cell growth (difference between total counts and spore counts) and toxin production were observed in control, apo-bLf-supplemented and iron-supplemented samples at both 24 and 48 h. However, little vegetative growth and no toxin were detected in the holo-bLf-supplemented samples (Figure 5a). The same was true following inoculation with *C. difficile* spores (Figure 5b), although toxin detection was delayed in the apo-bLf-supplemented samples compared with that in the control and iron-supplemented samples. Interestingly, the iron-supplemented samples inoculated with an overnight culture contained higher levels of spores at 48 h than the other samples, although this increased sporulation was not observed following initial inoculation with spores.

The samples inoculated with spores showed delayed toxin production compared with that in the samples inoculated in an overnight culture (4 RU versus 5 RU at 24 h for control samples), although the same final titre (5 RU) was observed at 48 h. This observation likely reflects the impact of spore germination, which is required before vegetative proliferation and toxin production can occur. The same delay would not be in place following inoculation of an overnight culture, as this culture will consist of a large proportion of vegetative cells.

## Discussion

We have investigated for the first time the effects of bLf, a natural bi-globular iron-binding glycoprotein with potent broad-spectrum antimicrobial activities,^9,22–24^ to prevent CDI *in vitro*. The triple-stage human gut model has been shown to produce results that correlate well with clinical observations.^25–33^ In the present study, the addition of holo-bLf delayed *C. difficile* vegetative cell growth and completely prevented toxin production. In contrast, *C. difficile* germination was seen much earlier in the apo-bLf model and was followed by rapid vegetative cell proliferation and detectable toxin production. These findings were reproduced in separate *in vitro* batch culture experiments. The presence of holo-bLf prevented vegetative cell proliferation and toxin production, whereas apo-bLf did not. Notably, the presence of excess iron alone did not prevent vegetative cell proliferation and toxin production. These additional studies suggest that the antimicrobial/inhibitory effect of holo-bLf is not likely to relate to iron toxicity. Furthermore, in comparison with apo-bLf, holo-bLf appeared to be more resistant to proteolytic degradation. These observations reflect similar findings *in vivo,* where iron-saturated bLf is more resistant to gut digestion.^34,35^ The exact mechanism or mechanisms by which holo-bLf exerts its increased antimicrobial activity remain elusive, but a possible explanation is that holo-bLf is more bioavailable (and resistant to degradation and proteolysis) than apo-bLf. A further possibility is that iron binding to bLf induces conformational changes that allow it to access the *C. difficile* cell wall. Following hydrolysis, positively charged lactoferrin-derived peptides (lactoferricin H and B) can interact with the negatively charged teichoic acid layer that surrounds the cytoplasmic membrane of Gram-positive bacteria.^36^ Incorporation of lactoferricin into the bacterial cytoplasm has been shown to inhibit DNA, RNA and protein synthesis.^37–39^ Whether the generation of lactoferricin might account for the activity of bLf against *C. difficile* growth and toxin production remains to be demonstrated. Alternatively, protein precipitation may account for the low levels of bLf in both models. The issue concerning protein precipitation was not explored, because of the complicated model set-up required. However, normally only a small fraction of proteins undergo precipitation due to lack of optimal environmental conditions or as a result of changes induced by components of the media. In general, gut microflora components were affected to a largely similar degree by clindamycin in both models. *Bifidobacterium* spp. were most clearly inhibited; reduced viable counts of *Bifidobacterium* spp. correlated with *C. difficile* spore germination and proliferation in all prior gut-model experiments with clindamycin.^32,33^ Furthermore, also reflecting previous observations,^40^ acetic, butyric and propanoic acid were the major SCFAs detected quantitatively, with smaller contributions from isobutyric, isovaleric and valeric acids. Clindamycin-induced disruption of gut-associated microbial communities was accompanied by a marked depletion of butyric acid SCFA levels. Similar results have been observed by Rea *et al.*,^41^ who reported that *C. difficile*-positive patients had a decrease in *Bacteroides*, *Prevotella* and *Bifidobacteria* and an increase in members of the Lactobacillaceae and Enterobacteriaceae families. Moreover, the relative abundance of butyric-acid-producing bacteria was significantly lower in patients with CDI and nosocomial diarrhoea.^42^ These findings suggest that the fermentation patterns or composition of SCFAs are closely related to bacterial composition and that variations in the bacterial flora and thus microbiota-derived metabolites may be more important in determining *C. difficile* susceptibility *in vivo*. It follows that as *Bifidobacteria* are normally present in high numbers in the human gut and are implicated in preventing establishment of pathogenic bacteria like *C. difficile*,^43^ their depletion may induce reduced production of SCFAs such as butyric acid, which has an important trophic effect on colonic epithelial cells.^44^ It is also possible that other compositional changes in specific butyrogenic bacterial species including *Clostridium leptum*, *Roseburia* spp., *Faecalibacterium prausnitzii* and *Coprococcus* spp. belonging to both the Firmicutes and Bacteroidetes phyla^45^ may have caused butyric acid depletion, particularly as we did not employ culture-independent high-throughput sequencing to dissect the role of antibiotics and lactoferrin on gut microbiota composition. Taken together, these studies suggest that individuals with sufficient levels of intestinal SCFAs, specifically butyric acid, may be less susceptible to CDI. Interestingly, commensal microbe-derived butyrate has been shown to induce the differentiation of colonic Foxp3-expressing regulatory T cells, which have a central role in the suppression of inflammatory and allergic responses.^46^ Mice fed a starch-rich butyrate-supplemented diet were more protected against the development of colitis compared with those fed a starch-rich diet without supplementation. Butyrate appeared to up-regulate the transcription of genes involved in differentiation of regulatory T cells in the gut by enhancing H3 acetylation.^46^

From a translational perspective, bLf has a proven good safety profile in pre-clinical animal studies and human clinical trials.^34,47–49^ The European Food Safety Authority, the FDA (USA) and the Therapeutic Goods Administration (Australia) have approved bLf for use in food, sports medicine and nutritional products. Oral delivery is the most common method for bLf administration. However, an appropriate delivery system is required to circumvent proteolytic digestion in the stomach and intestines and thus improve absorption and bioavailability within the gastrointestinal tract. Particulate carrier systems have been identified to protect bLf against proteolysis via encapsulation using pectin- and chitosan-modified liposomes and solid lipid particles.^50^ Nanoformulated iron-saturated bLf has been successfully tested for its antimicrobial efficacy. Orally fed, alginate gel-encapsulated ceramic nanocarriers loaded with iron-saturated bLf alone were more effective than ciprofloxacin therapy in salmonella-infected mice.^51^

The present study had a number of limitations. Firstly, given the nature, labour intensiveness and expense of the current experimental design, it was not possible to evaluate the effects of multiple strains of *C. difficile*, dose-ranging bLf concentrations or differing iron-saturation states of the bLf. We generated bLf iron-saturation states that represented each end of the spectrum (apo- and holo-forms), as such an approach is commonly cited. Although we tested only one concentration of bLf (5 mg/mL), we chose this concentration in line with previous randomized control trial data demonstrating its efficacy in significantly reducing the occurrence of antibiotic-associated diarrhoea in long-term care patients compared with placebo.^52^ Moreover, given the respective working volume of vessel 1 (280 mL), the total amount of bLf added per day of the experiment was 1400 mg. This dosage falls at the midpoint recommended by health and food companies for human consumption, which is <3 g of lactoferrin per day.^53^ It is largely unknown whether lactoferrin maintains its integrity and to what degree, and at what time it is hydrolysed into peptides in the human gastrointestinal tract. Moreover, the degradation of lactoferrin and the formation of bioactive peptides is highly dependent on individual variation in intraluminal composition.^54^ Factors such as gastric pH, age of the consumer and enzyme:substrate ratios seem to be important.^55^ Troost *et al*.^33^ showed that a major proportion (60%–80%) of bLf administered in a high dosage (15 mg/mL) survived passage through the stomach in adults. Therefore, we anticipate that it may be necessary to use higher oral dosages of bLf *in vivo* than those used in the gut model. It will thus be important to consider employing strategies to overcome insufficient bioavailability of lactoferrin through, for example, the use of pH sensitive polymer-coated drug delivery systems.^56^ For reasons cited earlier, we were also unable to evaluate the separate role of iron in the gut model set-up. Nevertheless, we did undertake comparative studies investigating the effects of control, apo-bLf, holo-bLf and excess iron in lactoferrin *in vitro* batch culture experiments. Furthermore, we did not investigate the stability of bLf through monitoring the derivatization products, including peptides such as lactoferricin. Finally, although this model is well validated and clinically reflective with regards to intestinal microbiota populations, it is limited in that it does not simulate host factors such as the immune response.^57^

Future investigations into the roles of apo-bLf, native bLf and holo-bLf *in vivo* are of clear importance in order to establish the role of bLf and its iron-binding status for the prevention and treatment of CDI. Whether treatment with bLf renders intestinal epithelia less susceptible to *C. difficile* toxins or microbial invasion remains to be investigated. Interestingly, bLf is effective in inhibiting cholera toxin-induced fluid accumulation in mice via abolition of toxin–receptor interaction.^58^ In future animal studies, we plan to perform bLf dose titration experiments and to evaluate the effects of alternative modes of drug delivery (for example, inter-gastric gavage, buccal deposition, intra-muscular). Follow-up work is also planned to test a range of lactoferrin saturation states in order to establish an optimal saturation level (initially 1%, 10%–15%, 40%–50% and >85%). In addition, we intend to test the effects of excess iron (iron oxide and iron oxide nanoparticles), liposomal-encapsulated iron-saturated bLf and purified lactoferricin in a murine model of CDI. Using this approach, the immunomodulatory, anti-inflammatory and cell-proliferative activities of lactoferrins^59,60^ may also be evaluated at the mucosal level. Laboratory studies will also be conducted to ascertain if the antimicrobial activity of bLf exerts beneficial effects on the structure and function of the murine intestinal microbiota. If our *in vitro* gut model observations are subsequently validated in the murine setting and are informative with regards to the optimal dosing strategy and delivery modality, we would aim to undertake clinical trials in the near future.

In conclusion, iron-enriched bLf is a comparatively inexpensive product that has proven to exhibit no adverse toxicity.^61^ The aforementioned panoply of features noted in this novel study indicate that holo-bLf might be a promising and attractive supplement to add to standard antimicrobial regimens against *C. difficile* and/or it could be used alone to potentially delay or inhibit *C. difficile* growth and toxin production. In addition, replenishing butyrogenic bacteria to allow *in situ* generation of butyric acid or other inhibitory compounds in the gut lumen may offer another novel therapeutic strategy for the treatment and prevention of CDI.

## Funding

This project was funded by an Early Starter Grant to T. M. from the Academy of Medical Sciences.

## Transparency declarations

In the past 2 years, C. H. C. has received research funding from Astellas Pharma Europe, Da Volterra and Cubist Pharmaceuticals and support to attend meetings from Astellas. M. H. W. has received honoraria for consultancy work, financial support to attend meetings and research funding from Astellas, AstraZeneca, Abbott, Actelion, Alere, AstraZeneca, Bayer, bioMérieux, Cerexa, Cubist, Da Volterra, Durata, Merck, Nabriva, Pfizer, Qiagen, Roche, Seres, Synthetic Biologics, Summit and The Medicines Company. All other authors: none to declare.

## Disclaimer

This is a summary of independent research funded by the National Institute for Health Research Biomedical Research Unit. The views expressed are those of the authors and not necessarily those of the NHS, the National Institute for Health Research (NIHR) or the Department of Health.
